# Nanoparticles containing aqueous seed extract of Syzygium cumini (npasc) protect against oxidized LDL particles *in vitro*

**DOI:** 10.1186/1758-5996-7-S1-A1

**Published:** 2015-11-11

**Authors:** Paula Eliete Rodrigues Bitencourt, Luana Mota Ferreira, Carolina dos Santos Stein, Manuela Sangoi, Laura Denardi, Raphaela Maleski Borges, Letícia Cruz, Rafael Noal Moresco, Sydney Alvez Hartz, Maria Beatriz Moretto

**Affiliations:** 1 Universidade Federal De Santa Maria, Santa Maria, Brazil

## Background

Diabetes mellitus (DM) is a heterogeneous group of metabolic disorders which affects over 10% of the world population. The diabetes-induced oxidized low-density lipoprotein (ox-LDL) can affect several components of the atherogenic process[1]. Nanodosage forms can provide advantages for herbal drugs, including increase of therapeutic index, improvement of stability and controlled delivery.

## Objectives

To evaluate the effect of NPASc and the major constituents of the extract (gallic acid, GA; chlorogenic acid, CA; rutin, R) on the levels of lipoperoxidation of ox-LDL particles by AAPH, *in vitro*.

## Materials and methods

LDL was isolated from human serum (n=6)[2]. LDL isolated samples were incubated with/without NPASc, GA, CA and R (0.1; 0.25; 0.5 and 1 mg/mL) at 37°C for 30 min. At the end of incubation, the samples were oxidized in the presence of 20 µM AAPH, for 4 h at 37°C. The oxidation was estimated by measuring the thiobarbituric acid reactive substances (TBARS, nmol MDA/mg of protein)[3]. N° of the Ethic Committee (0049.0.243.000-08).

## Results

Our results demonstrate that NPASc and the compounds protected LDL particles from the oxidation by AAPH, demonstrating that the known antioxidant activity of S. cumini was maintained. GA and CA showed a significant antioxidant activity, although less than that observed with NPASc; R had a lower effect when compared to other groups. This result confirms the possible antiatherogenic potential of the extract.

## Conclusion

NPASc could act to lower the ox-LDL presence in the circulation, reducing the number of proatherogenic potentials thus avoiding the formation of atherosclerotic lesions.

**Figure 1:**
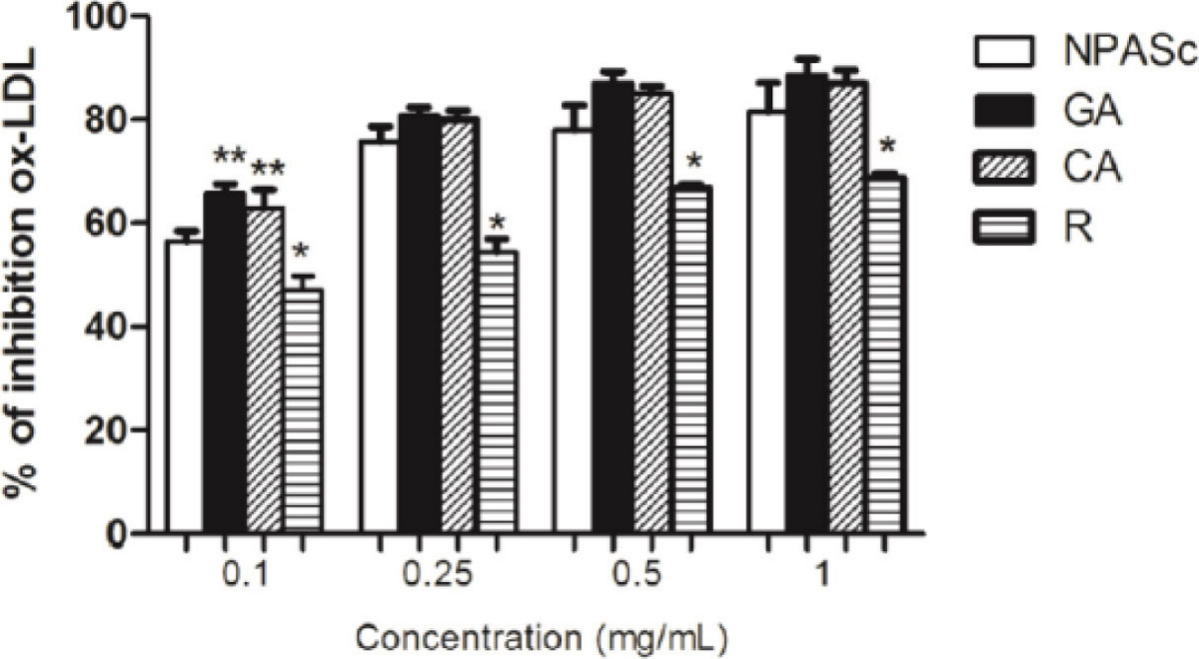
**NPASc, GA, CA and R activities against ox-LDL in the AAPH assay.** Data are mean±S.E.M (n=6). Differences among compounds within the same concentration: *p<0.05 compared to all; **p<0.05 compared to NPASc
